# Public availability of information from WFME-recognized accreditation agencies

**DOI:** 10.1186/s12960-021-00621-z

**Published:** 2021-06-29

**Authors:** Kahlo Baniadam, Zakia Arfeen, Mohammed Ahmed Rashid, Ming-Jung Ho, Sean Tackett

**Affiliations:** 1 Georgetown University School of Medicine, Washington, USA; 2 University College London, London, USA; 3 Georgetown University Medical Center, Washington, USA; 4 Johns Hopkins Bayview Medical Center, 5200 Eastern Ave, MFL Center Tower Suite 2300, Baltimore, MD 21224 USA

**Keywords:** Medical school accreditation, Transparency, Quality assurance

## Abstract

The World Federation for Medical Education (WFME) Recognition Programme was created to ensure the comparability of medical school accrediting agencies, so that the schools accredited by those agencies would have similar educational quality. WFME explicitly values transparency and has recognition criteria that relate to agencies making information publicly available. Our study examined 20 WFME-recognized agencies’ transparency by reviewing agency websites for 27 information elements related to accreditation standards, procedures, and processes. We contacted agencies as needed for information that we could not find on their websites. We were only able to retrieve additional information from 3 of the 12 agencies that we attempted to contact. We found that while 12 agencies had over 90% of expected information elements available, 6 agencies had less than 50%. Our findings illustrate barriers for those who wish to better understand medical school accreditation in some regions and raise questions about how comparable WFME-recognized agencies are.

## Introduction

Historically, international medical graduates (IMGs) have constituted a quarter of practicing physicians in the United States (U.S.) [1]. Before being permitted to care for patients in the U.S., all IMGs must be certified by the Educational Commission for Foreign Medical Graduates (ECFMG). ECFMG has requirements that IMGs must meet for certification, which include passing a series of standardized exams and demonstrating graduation credentials (e.g., diploma) from a medical school [2]. ECFMG applicants come from close to 2000 different medical schools, across over 150 countries, which are under the purview of approximately 100 different accrediting bodies [3]. Given the rapid growth in medical schools and uneven practices of accrediting authorities worldwide, in 2010, the ECFMG announced an Accreditation Requirement, where, starting in 2023, all IMGs applying for ECFMG certification would be required to have attended a medical school accredited by an agency that meets the criteria of the World Federation for Medical Education (WFME) Recognition Programme [4]. ECFMG has since adjusted the deadline to 2024 due to the COVID-19 pandemic [5].

WFME describes on its Recognition Programme website that the “Recognition Programme delivers an independent, transparent and rigorous method” [6]. Among WFME’s recognition criteria for accrediting authorities are that “6. The agency makes publicly available the accreditation standards,” and “20.1. The agency makes publicly available information on accreditation policies and procedures” [7]. ECFMG leaders have similarly stated that “Accreditation fosters quality improvement, transparency and, above all, public trust.” [2]. Other authors have argued for greater transparency in accreditation processes to increase awareness among educators about its value, foster collaboration and learning among medical schools, and ensure the public remains informed [8].

Our study team initially wanted to compare standards, policies, and procedures across WFME-recognized agencies to determine which elements varied across contexts and which were more universal. Based on WFME’s stated principles and its specific recognition criteria, we assumed that agencies that had completed the recognition process would have made this information publicly available. However, in attempting to pursue our original project, we struggled to find information across agencies. Instead, we decided to systematically characterize the availability of agencies’ information as a marker of their transparency.

## Methods

### Definition of transparency and public availability

A priori we decided that meeting WFME recognition criteria by having information “publicly available” would be an acceptable level of transparency. We decided that information would be most “publicly available” if it were accessible by anyone who went to the agency’s official website, although we also decided that receiving information within a reasonable time frame of 6 weeks after requesting it could constitute availability.

### WFME-recognized agencies

At the time of study design, there were 21 WFME-recognized agencies. We excluded one agency because one of our authors had a potential conflict of interest in evaluating the agency. From April to July 2020, two authors independently reviewed each of the remaining 20 agencies’ official websites (obtained from the WFME website) [9] for 27 information elements. These elements were adapted from a published framework describing 26 “components” of accreditation that were based on WFME recognition criteria [10] and a pilot review of a subset of agency websites. We modified the components of accreditation framework by eliminating 2 information elements about accreditation standards that did not have relevance to our study aims (i.e., standards format, method of dissemination of standards) leaving us with 24 essential information elements that would have been required for WFME recognition. We also added 3 information elements that were not detailed in WFME recognition criteria (i.e., publishing school reports, agency fees for accreditation review, and fees for special services) but we felt would be of interest to the medical education community given their public and financial implications and variable practices among accrediting agencies. Aspects of each information element were recorded as being present (as website text or downloadable document) or not present. We felt that this dichotomous cutoff (i.e., an element was present or not) was more reliable than attempting to make judgments about the quality of information present. WFME requires documents to be in English for its Recognition Programme [11], and our team comprised native English speakers, so information elements were only recorded as being present if we were able to verify this in an English language translation on the website or in an English language document. For any of the 24 essential information elements that were not present in English after checking every page on the agency site, we attempted to contact the agency through all possible official contact mechanisms (e.g., email addresses, web inquiry forms), asking for the specific essential information elements that we were unable to find (we did not request information for the 3 supplemental elements (i.e., publishing school reports, agency fees for accreditation review, and fees for special services)). We attempted to clarify requests as needed in correspondence with agencies. A reminder email was sent if there was no reply to our inquiries. We stopped inquiring if no reply was received after a total of 6 weeks.

### Data analysis and ethical approval

Of the 20 agencies examined, 2 agencies were excluded from data analyses because one agency’s website provided no information about the agency and no means to ask for additional information, and another agency’s site gave an error message during the data collection period. One of the remaining 18 WFME-recognized authorities provided different information based on the regions that it covered and was treated as two separate agencies in analysis, leaving us with a total of 19 agencies. For analyses by each information element across all agencies, we tabulated descriptive statistics for all 27 accreditation information elements across the 19 agencies. For summary calculations by agency, we provide data for the 24 essential information elements required by WFME. Our study protocol was reviewed by a Johns Hopkins Medicine Institutional Review Board and deemed non-human subjects research.

## Results

Across all 27 information elements related to accreditation standards, policies, and procedures, each element had a mean of 12.7 (67%) agencies that made it publicly available (Table 1). Information was most commonly available as a document on the agency’s website (68%).

Additional information was needed from 12 agencies after website review. One agency had no means of requesting information (i.e., no contact email or web inquiry form). We contacted 11 agencies and received the information we requested from 3 agencies.

Among all 27 information elements across all 19 agencies, the source that provided the agency with its regulatory authority was available for all agencies (n = 19, 100%), with aspects of the agency’s composition being available second-most commonly (n = 17, 89%). Fees for accreditation services were least commonly reported (Table 1).

**Fig. 1.**
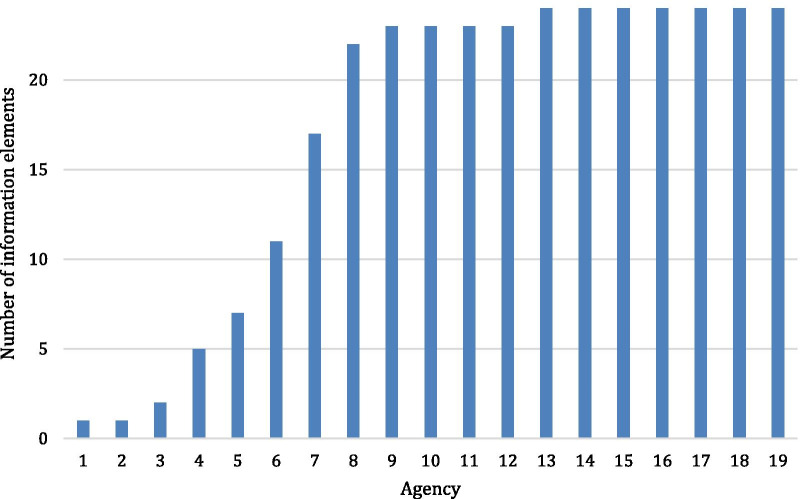
Distribution of availability of 24 information elements for 19 accreditation agencies^a^. ^a^As described in methods, one agency was counted as two agencies because information was shared separately according to region that it covered. Two agencies had no information available and are not shown in this Figure

Across agencies, the median percentage of the 24 essential information elements that were publicly available was 96% (Fig. 1). However, while there were 12 agencies that reported over 90% of expected information elements, 6 agencies reported less than half (Fig. 1).

## Discussion

Accreditation of medical schools exists to assure the public that physicians have received the education required for them to provide safe patient care. Transparency of accreditation policies and practices permits broad stakeholder awareness and engagement that can improve accreditation processes and optimize quality assurance for the public. Transparency is also espoused by ECFMG and WFME [2, 6]. Many WFME-recognized agencies make abundant information available. However, a surprising number of agencies do not make information readily available on their websites, and we found that agencies rarely responded effectively to requests for information.

During our data collection, we counted an information element as being present when a minimal amount of information was found. For example, if an agency document mentioned site visits and that a team of five visitors would tour schools, we reported this as satisfying both the site visit process and team characteristics elements in the site visit category. The lowest transparency agencies accordingly had essentially no information related to the information elements that we expected to find. We also excluded two agencies from our quantitative analysis because their websites were not accessible at the time data was collected. While one agency’s website had come online by the time this manuscript was being prepared, the other continued to offer no option to learn more about the agency or its processes. Therefore, our quantitative data may overestimate the availability and the quality of the information provided by agencies. Using this conservative approach, we still found some agencies to have very limited information available and one agency with no information available.

We did not systematically document the quality of information organization on websites, but we found many websites difficult to navigate in their organization and language. Certain websites had portions in the domestic language of the agency with documents that were in English. This presented challenges to our team’s identification of information elements, but also presumably could make it difficult for local stakeholders who would need to review documents in English when that may not be the language they use regularly. In addition, while it may be reasonable for agencies to make information available upon request, rather than include all information on their websites, the fact that so few agencies responded to requests for additional information is concerning. The limited information that is made available, difficult navigation of websites by low transparency agencies, and lack of response to requests presented significant barriers for our team, which was composed of individuals who were already familiar with accreditation and actively looking for specific information. We suspect that for students, educators, and members of the public who may be unfamiliar with accreditation or less motivated, these same barriers could make an understanding of accreditation more difficult and potentially inaccessible.

The WFME Recognition Programme was developed to ensure that the quality of accreditation processes was comparable across agencies, thereby ensuring that medical school graduation credentials for ECFMG applicants had similar meaning across jurisdictions. While not all WFME recognition criteria must be met to achieve recognized status, a significant proportion of agencies did not appear to be meeting two recognition criteria. Not all 24 of the information elements that we classified as essential may be necessary for agencies to share with the public, but we were unable to review any documentation of an agency’s accreditation standards—referenced specifically in a WFME recognition criterion—for over a quarter of agencies. It is possible that the WFME criteria related to the public availability of information were considered during the recognition process in light of a certain agency’s local level of acceptability of information-sharing or the feasibility of the agency to make information publicly available given its resource constraints. However, WFME does not share on its website information about its reviews of agencies, which precluded our study from describing how these criteria were considered and applied during WFME review. If recognition criteria were to be applied inconsistently for agencies, then the schools that they accredit may not have comparable quality across settings.

Our study indicates that most of the first group of WFME-recognized agencies make abundant information available to the public, but that some present significant barriers for stakeholders to engage more with accreditation due to a lack of transparency. The variation that we found also raises questions about the comparability of WFME-recognized agencies that warrant further exploration as the WFME Recognition Programme expands and the ECFMG 2024 deadline draws near.

**Table 1.** Presence and form of availability of 27 information elements by 19 accrediting agencies, n (%)

		Total	Document	Website text	Email request
Overall means		12.7 (67%)	8.5 (68%)	3.3 (25%)	0.9 (7%)
Agency structure	Internal aspects (e.g., membership qualifications and number)	17 (89%)	3 (18%)	14 (82%)	0 (0%)
	External aspects (e.g., source of authority, relationships with other organizations)	19 (100%)	3 (16%)	16 (84%)	0 (0%)
Agency internal policies and resources	Conflicts of interest management	14 (74%)	12 (86%)	2 (14%)	0 (0%)
	Record keeping	12 (63%)	10 (83%)	1 (8%)	1 (8%)
	Financial, space, material, and human resources management	11 (58%)	7 (64%)	3 (27%)	1 (9%)
Standards for schools^a^	Content (e.g., topics covered, rigor, quality)	14 (74%)	12 (86%)	1 (7%)	1 (7%)
	Process for development and/or revision (e.g., stakeholder input)	13 (68%)	10 (77%)	2 (15%)	1 (8%)
Self-study	Guidance provided by agency (e.g., templates, other support or consultation)	14 (74%)	10 (71%)	2 (14%)	2 (14%)
	School process (e.g., participants, cost, duration)	14 (74%)	12 (86%)	1 (7%)	1 (7%)
Site visit by reviewers	Process (e.g., preparation, duration, activities)	14 (74%)	10 (71%)	2 (14%)	2 (14%)
	Site visit team characteristics (e.g., demographics, training, expertise)	13 (68%)	8 (62%)	4 (31%)	1 (8%)
Site visit team’s report for agency	Content (e.g., synthesis of findings, recommendations)	13 (68%)	9 (69%)	2 (15%)	2 (15%)
	Process (e.g., writing, medical school review)	14 (74%)	9 (64%)	3 (21%)	2 (14%)
Agency’s evaluation of school^a^	Methods of reviewing information and communicating internally	14 (74%)	10 (71%)	3 (21%)	1 (7%)
	Criteria and/or process (e.g., voting) for making summary decisions	13 (68%)	10 (77%)	2 (15%)	1 (8%)
	School reports publicly available^b^	6 (32%)	2 (33%)	4 (67%)	0 (0%)
Agency’s summary decision and recommendations	Types of summative decisions (e.g., full accreditation, probation, withdrawal)	14 (74%)	10 (71%)	3 (21%)	1 (7%)
	Feedback for schools (e.g., recommendations for improvement, positive feedback)	12 (63%)	10 (83%)	1 (8%)	1 (8%)
	External communications (e.g., public transparency, information for licensing bodies)	14 (74%)	10 (71%)	2 (14%)	2 (14%)
	Managing appeals (e.g., timing, process)	14 (74%)	9 (64%)	4 (29%)	1 (7%)
Follow-up and monitoring	Follow-up intervals	12 (63%)	10 (83%)	2 (17%)	0 (0%)
	Methods of data collection (e.g., school self-report, revisiting site)	13 (68%)	11 (85%)	2 (15%)	0 (0%)
	Complaints	14 (74%)	8 (57%)	6 (43%)	0 (0%)
New schools^a^	Process of receiving initial accreditation (e.g., agency support, accreditation stages)	13 (68%)	10 (77%)	2 (15%)	1 (8%)
	Differences in accreditation procedures for new versus existing schools	11 (58%)	9 (82%)	0 (0%)	2 (18%)
Fees	Fees for accreditation^b^	7 (37%)	4 (57%)	3 (43%)	0 (0%)
	Fees for special services^b^	3 (16%)	2 (67%)	1 (33%)	0 (0%)

^a^Some agencies refer to “programs” rather than “schools” as to what they accredit. Data include program or school based on agency terminology

^b^Denotes one of the three items that were not felt to be mandatory and were not requested from agencies if not found on agency websites

## Data Availability

All data for this study were captured on web pages or may be requested from agencies.
